# Atomic force microscopic image data of botulinum neurotoxin complexes with different molecular sizes

**DOI:** 10.1016/j.dib.2019.104193

**Published:** 2019-06-25

**Authors:** Shin-Ichiro Miyashita, Yoshimasa Sagane, Takamasa Uchino, Shura Karatsu, Keita Hosoya, Ihsun Huang, Koichi Niwa, Toshihiro Watanabe, Yoichi Niimura, Tomonori Suzuki

**Affiliations:** a Department of Food and Cosmetic Science, Graduate School of Bioindustry, Tokyo University of Agriculture, 196 Yasaka, Abashiri, Hokkaido 099-2493, Japan; b Department of Food, Aroma and Cosmetic Chemistry, Faculty of Bioindustry, Tokyo University of Agriculture, 196 Yasaka, Abashiri, Hokkaido 099-2493, Japan; c Department of Molecular Microbiology, Faculty of Life Sciences, Tokyo University of Agriculture, 1-1-1 Sakuragaoka, Setagaya-ku, Tokyo 156-8502, Japan; d Department of Health and Nutrition, Faculty of Human Science, Hokkaido Bunkyo University, 5-196-1 Kogane-chuo, Eniwa, 061-1449, Japan

**Keywords:** *Clostridium botulinum*, Botulinum toxin complex, Atomic force microscopy, Protein structure, AFM, atomic force microscopy, TC, toxin complex, BoNT, botulinum neurotoxin

## Abstract

This data article provides atomic force microscopy (AFM) amplitude images of botulinum toxin complex (TC) molecules produced by *Clostridium botulinum* serotype D strain. *C. botulinum* produces different-sized TC molecules, such as a complex of botulinum neurotoxin and nontoxic nonhemagglutinin proteins (M-TC) and complex of M-TC and hemagglutinin subcomplex (L-TC). In this data article, the M and L-TC produced by serotype D strain 4947 were imaged by AFM. The M-TC molecule had a globular structure with a 30.5-nm diameter and a 2.1-nm height, while the L-TC molecule had a distinct structure in which several spheres were connected to a globular structure that was 40.7 nm in diameter and 3.5 nm in height.

**Table:** Specifications table

Subject area	Biochemistry
Specific subject area	Protein Chemistry
Type of data	Images Table
How data was acquired	Atomic force microscopy using MFP-3D (Asylum Research, Santa Barbara, CA). Data analysis was conducted using IGOR Pro 5.02 software (Wave Metrics Inc., Portland, OR).
Data format	Raw and analyzed
Parameters for data collection	Two types of TC (M-TC and L-TC) were purified from culture supernatant of *C. botulinum* serotype D strain 4947.
Description of data collection	Atomic force microscopic images of purified botulinum TCs were acquired under tapping mode.
Data source location	Abashiri, Japan
Data accessibility	Data are presented in this article

**Table:** 

**Value of the data** • The first images of botulinum TC acquired here by AFM would be useful for investigating the structure and function of TC, and the stable and effective transport of the toxin into human and animals, thereby providing an understanding of the pathogenicity of botulinum neurotoxin. • The AFM images of the botulinum TCs improve the understanding of the molecular shape of the complexes. • The AFM images of two different forms of TCs can be used to assess molecular size, which will enable us to examine the transport of the toxin.

## 1 Data

**Fig. 1:**
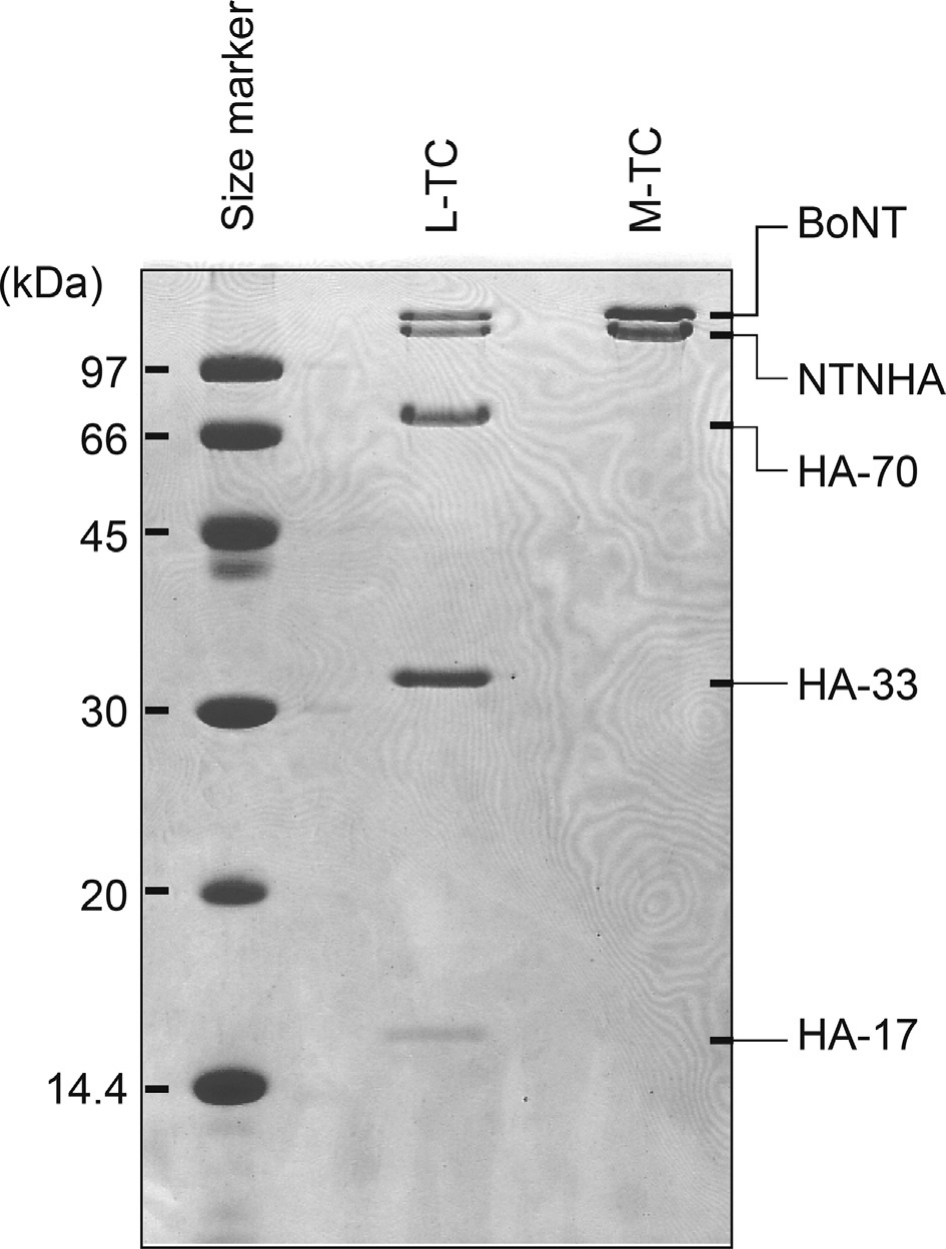
Banding profiles of M-TC and L-TC on SDS-PAGE. Molecular masses of size marker proteins are indicated on the left side, while component proteins from each TC are indicated on the right side of the gel.

**Fig. 2:**
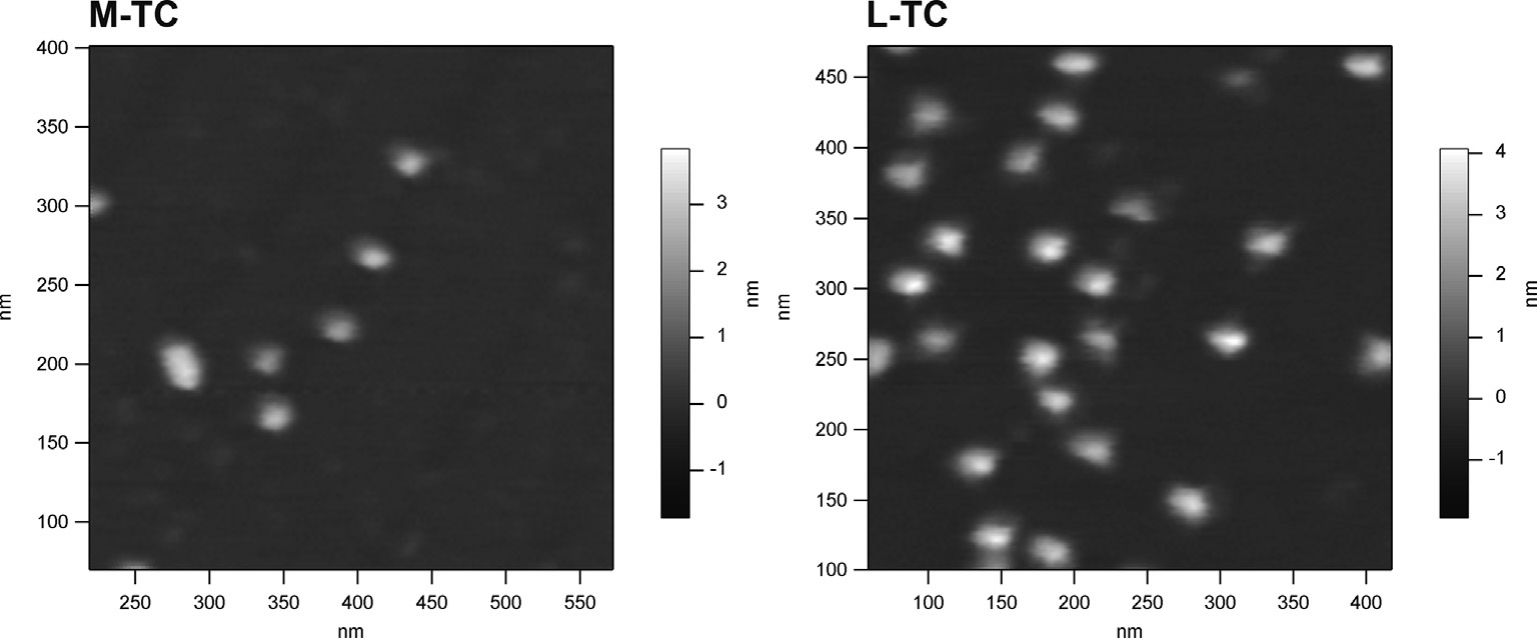
AMF amplitude images for M-TC and L-TC deposited on mica plate.

This data article provides three sets of data. The purities of the isolated complexes of botulinum neurotoxin and nontoxic nonhemagglutinin proteins (M-TC) and complex of M-TC and hemagglutinin subcomplex (L-TC) were assessed by sodium dodecyl sulfate-polyacrylamide gel electrophoresis, as shown in Fig. 1. The purified TCs were subjected to atomic force microscopy (AFM) analyses by tapping mode using an SSS-NCH probe. Fig. 2 shows the AFM amplitude images for the M-TC and L-TC deposited onto the mica plate. The diameters and heights of the TCs assessed by AFM are shown in Table 1.

**Table 1:** Diameter and height of M-TC and L-TC estimated by cross-section analysis of AFM images.

L-TC		M-TC	
Diameter (nm)	Height (nm)	Diameter (nm)	Height (nm)
40.7	3.5	30.5	2.1

## 2 Experimental design, materials, and methods

### 2.1 Design

*Clostridium botulinum* is classified into seven serotypes, A–G, based on immunological differences in botulinum neurotoxin (BoNT) produced by the strains. BoNT exists as a part of two different-sized toxin complexes (TCs) in which a single molecule of BoNT binds to a single nontoxic nonhemagglutinin molecule yielding M-TC, and as the complex (L-TC) of M-TC and hemagglutinin subcomplex (a complex of three HA-70 molecules, three HA-17 molecules, and six HA-33 molecules) [1]. Here, we purified two types of TCs, M-TC and L-TC, from the culture supernatant of the *C. botulinum* serotype D strain and performed AFM analysis.

### 2.2 Materials

*C. botulinum* serotype D strain 4947 was used to produce botulinum TC.

### 2.3 Production and purification of botulinum TCs

The botulinum TC was produced as described previously [2] with minor modifications [1]. Briefly, TCs in the culture supernatant were precipitated with ammonium sulfate at 60% (w/v) saturation and the resulting precipitate was dialyzed against 50 mM acetate buffer (pH 4.0) containing 0.2 M NaCl. The sample solution was applied to a TOYOPEARL SP-650S (Tosoh, Tokyo, Japan) cation-exchange column (1.6 × 40 cm) equilibrated with dialysis buffer. The TCs bound to the resin were eluted over a linear gradient of NaCl (0.2–0.8 M). The M- and L-TC fractions were pooled separately, concentrated, and further purified with a HiLoad 16/60 Superdex 200 pg gel-filtration column (GE Healthcare, Little Chalfont, UK 1.6 × 60 cm) equilibrated with 50 mM acetate buffer (pH 5.0) containing 0.15 M NaCl. The TC fraction was then applied to a Mono S HR5/5 cation-exchange column (GE Healthcare UK; 0.5 × 5 cm) equilibrated with 50 mM acetate buffer (pH 5.0) and eluted over a linear gradient of NaCl (0–0.5 M). The purities of the M- and L-TC were evaluated using native polyacrylamide gel electrophoresis (PAGE) and sodium dodecyl sulfate (SDS)-PAGE.

### 2.4 PAGE

SDS-PAGE was performed as described by Laemmli [3] with a 15% polyacrylamide gel with 2-mercaptoethanol. PAGE under non-denaturing condition was carried out as described by Davis et al. [4] at pH 8.8 using a 5–12.5% polyacrylamide linear gradient gel. The separated protein bands were stained with Coomassie Brilliant Blue R-250.

### 2.5 AFM analysis

Data were collected in air using an SSS-NCH probe (tip radius < 5 nm, nominal resonant frequency 300–350 kHz, nominal spring constant 42 N/m) on an MFP-3D atomic force microscope (Asylum Research, Santa Barbara, CA, USA) in tapping mode. A 50-μL droplet of 0.5–5 nM M- or L-TC was deposited on 10-mm^2^ mica and allowed to adsorb for 10 min. After rinsing with ultra-pure water, the mica was dried at 40 °C for 15 min, and then subjected to AFM. In addition, the length and width of the proteins were validated using the cross-section analysis in the IGOR Pro 5.02 software (Wave Metrics Inc., Portland, OR) on the MFP-3D AFM apparatus. The height was defined as the measure of the vertical distance from the bottom to the top of the protein on mica and the width as the measure of the diameter of the protein.
